# Triple-layered core-shell fiber dressings with enduring platelet conservation and sustained growth factor release abilities for chronic wound healing

**DOI:** 10.1093/rb/rbae034

**Published:** 2024-03-23

**Authors:** Simin Lai, Tingbin Wu, Chenxi Shi, Xiaojing Wang, Pengbi Liu, Lihuan Wang, Hui Yu

**Affiliations:** Guangdong–Hong Kong Joint Laboratory for New Textile Materials, School of Textile Science and Engineering, Wuyi University, Jiangmen 529020, China; Guangdong–Hong Kong Joint Laboratory for New Textile Materials, School of Textile Science and Engineering, Wuyi University, Jiangmen 529020, China; Guangdong–Hong Kong Joint Laboratory for New Textile Materials, School of Textile Science and Engineering, Wuyi University, Jiangmen 529020, China; Jiangmen Central Hospital, Jiangmen 529030, China; Guangdong–Hong Kong Joint Laboratory for New Textile Materials, School of Textile Science and Engineering, Wuyi University, Jiangmen 529020, China; Guangdong–Hong Kong Joint Laboratory for New Textile Materials, School of Textile Science and Engineering, Wuyi University, Jiangmen 529020, China; Guangdong–Hong Kong Joint Laboratory for New Textile Materials, School of Textile Science and Engineering, Wuyi University, Jiangmen 529020, China

**Keywords:** chronic wounds, bioprinting, platelet-rich plasma, sustained release, long-term storability

## Abstract

Platelet-rich plasma (PRP) is one of the most popular biomaterials in regenerative medicine. However, the difficulties encountered in its preservation, and the requirement for on-demand preparation severely limit its application. In addition, its rapid degradation in the wound microenvironment makes the sustained release of growth factors impossible and finally reduces the therapeutic effect on chronic wounds. Here, a multifunctional dressing based on triple-layered core-shell fibers for loading and enduring preservation of PRP was developed using a one-step coaxial bioprinting technique combined with freeze-drying. The platelets were effectively dispersed and immobilized in the core layer of the fiber, leading to a sustained release of growth factors from the PRP. The rate of release can be controlled by adjusting the triple-layered core-shell structure. Simultaneously, the triple-layered core-shell structure can reduce the deactivation of PRP during freezing and storage. The experimental findings suggest that PRP exhibits sustained activity, facilitating the process of wound healing even after a storage period of 180 days. Furthermore, the protective mechanism of PRP by the triple-layered core-shell fiber was investigated, and the conditions for freeze-drying and storage were optimized, further enhancing the long-term storability of PRP. As a result, the multifunctional core-shell fiber dressings developed in this study offer a novel approach for sustained growth factor release and the enduring preservation of active PRP.

## Graphical Abstract

**Figure:**
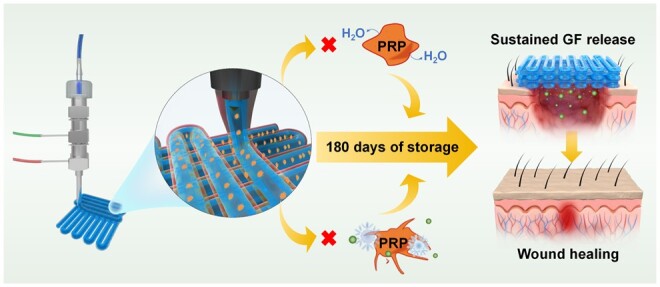

## Introduction

The skin, which is the largest organ of the human body, serves as the primary defense mechanism against an invasion of external microorganisms [1]. Extended exposure to the external environment during daily activities compromises the integrity of the skin, making it one of the most susceptible organs. The issue of wound management, particularly for chronic ulcers, has become a substantial worldwide public health concern. Extended treatment durations, excessive expenses and often uncertain therapeutic outcomes characterize these wounds, imposing a significant financial burden on governments worldwide [2]. The clinical approach to wound treatment still relies on traditional wound dressings such as gauze, bandage and foam. However, owing to the limited functionality of these dressings, the wound healing cannot be achieved [3]. To address these issues, various biomaterials have been designed and developed to promote wound healing [4–6]. Recently, platelet-rich plasma (PRP), enriched with platelets, has emerged as a novel biomaterial.

PRP is obtained through the centrifugal concentration of human or animal whole blood. Upon activation, PRP releases various growth factors (GFs), such as vascular endothelial growth factor (VEGF), platelet-derived growth factor (PDGF) and epidermal growth factor (EGF), among others. These factors can stimulate granulation tissue formation, angiogenesis and epithelialization, ultimately fostering wound healing. PRP has found widespread applications in various regenerative fields, including orthopedic regeneration, medical aesthetics, hair regeneration and skin regeneration [7–10]. Generally, PRP is processed into PRP-gel (PRP-gel) through the addition of Ca^2+^ or thrombin, and subsequently administered at targeted sites. However, this method has certain limitations. Pure PRP-gel is vulnerable to a range of physical and chemical factors, including temperature, pressure and the instability of fibrin. This susceptibility can lead to rapid degradation of PRP within the wound microenvironment, which is rich in proteases. As a consequence, growth factors (GFs) are swiftly released into the tissue fluid, impeding sustained and prolonged release. Additionally, the difficulties in preserving PRP-gel, which requires immediate preparation and use, contribute to a higher frequency of PRP administration. This increased treatment frequency often leads to significant discomfort for patients [11–14]. Therefore, enhancing the long-term storage stability of PRP and the efficient release of GFs is a key challenge in PRP therapy. Zhang *et al*. [15] developed an extracellular matrix mimic hydrogel for PRP encapsulation, achieving sustained GFs release by inhibiting PRP enzymatic degradation. Ho *et al*. [16] mixed PRP with a carbohydrate to shield cells from oxidative damage, thereby extending the storage time of PRP to as long as 14 days. Recently, Rao *et al*. [17] developed a hyaluronic acid gel matrix to load PRP while ensuring the stability of platelets during storage. Presently, most PRP-related hydrogel dressings primarily address sustained growth factors release, yet their efficacy is limited by insufficient long-term storage stability. Hence, the development of a multifunctional dressing enabling sustained growth factors release and long-term PRP storage holds considerable clinical significance.

**Figure 1.**
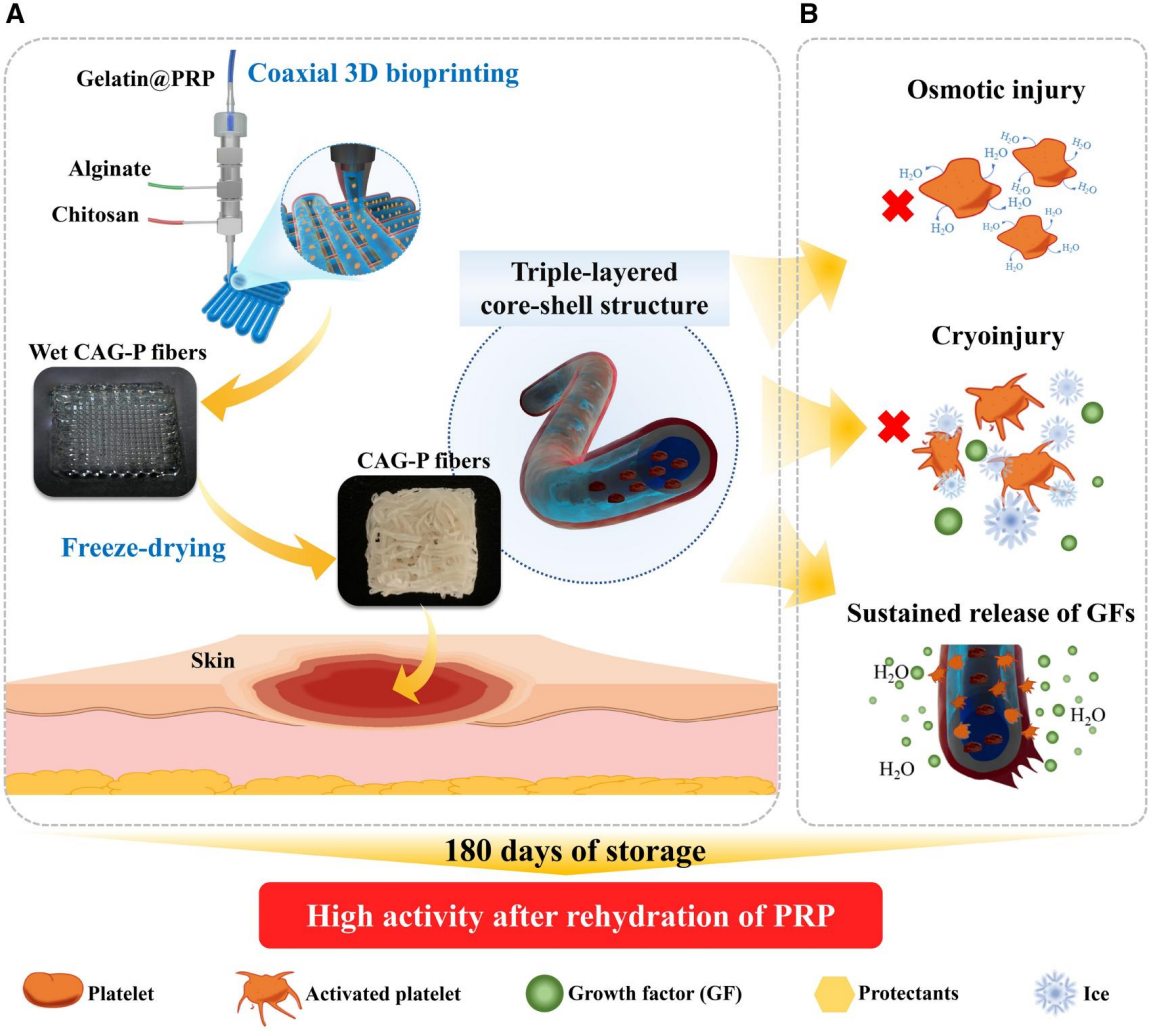
(**A**) Schematic representation of CAG-P fiber dressing preparation and (**B**) the mechanism of triple-layered core-shell structure effecting on GFs sustained release and PRP long-term storage.

Biomedical wound scaffolds that mimic the natural extracellular matrix (ECM) are designed as wound healing material [18]. Among various forms of scaffolds, nanofibers and hydrogels are frequently used as skin substitutes [19]. In recent years, hydrogels prepared through three-dimensional (3D) bioprinting technology have been widely applied in drug research [20], implants [21], tissue engineering [22] and other fields. Building upon these advancements, a fiber dressing referred to as the triple-layered core-shell structure was designed. This innovative design is achieved through a combination of one-step coaxial 3D bioprinting and freeze-drying techniques, utilizing chitosan/alginate/gelatin as the bioink (Figure 1), and thus the obtained fibers are abbreviated as CAG fibers. Chitosan (CS), known for its biocompatibility, biodegradability and antibacterial properties, plays a pivotal role in promoting fibroblast proliferation, vascular network formation, and the periodic deposition of collagen fibers [23–26], thereby serving as the shell layer of the fiber. Alginate (AL) possessing favorable biocompatibility and liquid absorption properties, serves as the intermediate layer of the fiber to facilitate wound healing by creating a moist environment [27–29]. Furthermore, the presence of sodium alginate enables rapid cross-linking by bivalent or polyvalent cations, facilitating the 3D printing process. Gelatin, an essential constituent of ECM, promoting cell adhesion, differentiation and proliferation [30–32], is used as the core layer to load PRP. The fibers loaded with PRP, termed CAG-P fibers, encompass not only fundamental properties like exceptional water absorption, retention, antibacterial activity and biocompatibility, but also integrate multiple functions such as PRP loading and long-term storage, along with sustained release of growth factors. The multilayer structure of the fiber ensures that PRP is effectively dispersed and immobilized in the fiber core layer, thereby extending the release of growth factors. Additionally, the fiber dressing can preserve PRP for an extended period, with PRP retaining a certain level of activity even after 180 days of storage, thus accelerating wound healing. This research addresses the clinical challenge of immediate synthesis and utilization of PRP, providing a potential solution to the associated preservation challenges.

## Experimental section

### Materials

Chitosan was procured from China National Pharmaceutical Group Chemical Reagent Co., Ltd (China). Alginate was obtained from Sigma-Aldrich (USA). Gelatin was procured from China Macklin Company (China). Anhydrous calcium chloride was sourced from Xilong Scientific Co., Ltd (China). PRP was acquired from Zhuhai Baishitong Biotechnology Co., Ltd (China).

### Preparation of PRP-gel, CAG fibers and CAG-P fibers

#### Preparation of PRP-gel

To prepare PRP-gel, thrombin (2 KU) was mixed with 1 ml of a 10 wt% calcium chloride (CaCl_2_) solution to obtain an activation solution, and then 30 μl of PRP was mixed with 300 μl of the activation solution.

#### Preparation of wet CAG fibers and CAG-P fibers

A coaxial nozzle comprising three microchannel layers was used by implementing a one-step coaxial microfluidic 3D bioprinting method. Stable triple-layered core-shell fiber hydrogels were formed by adjusting the flow rates of the outer phase channel (outer layer, 1 wt% CS), middle phase channel (middle layer, 2 wt% sodium alginate) and inner phase channel (inner layer, 1 wt% gelatin) via 3D deposition. Then, wet CAG fibers were solidified using 10 wt% CaCl_2_. The wet CAG-P fibers were obtained by loading PRP into the inner phase channel (the volume ratio of gelatin solution to PRP was 7:1), and the remaining preparation methods were the same as for the wet CAG fibers.

#### Preparation of CAG fibers and CAG-P fibers

The prepared wet CAG fibers and CAG-P fibers were rapidly frozen in liquid nitrogen for 10 min. It was then transferred to a freeze-dryer and dried in a low vacuum (0.043mbar) chamber at ice condenser temperature of −80°C for 48 h to obtain CAG fibers and CAG-P fibers, and stored in a vacuum at −20°C for a long time.

### Morphological analysis

Field emission scanning electron microscopy (FE-SEM; Sigma 500, Zeiss, Germany) was employed to observe and analyze the morphology of fiber dressings, fiber dressings after degradation, and the PRP loaded in the CAG fibers. The accelerating voltage was set at 5 kV. The PRP in CAG-P fibers and the distribution of particles within PRP were observed using field emission transmission electron microscopy (FE-TEM; JEM-F200, Japan) operating at a voltage of 200 kV.

### Swelling experiment

The initial weight (*m_0_*) of the dry CAG fiber dressing was measured and recorded in grams. Subsequently, the fiber dressing was immersed in a 10 ml phosphate buffer saline (PBS) maintained at 37°C. The fiber dressing was taken out at designated time intervals and excess solution on the surface was absorbed using filter paper. The weight of the swollen sample (*m_1_*) was measured. The swelling ratio (*S_w_*) was calculated using the formula shown in Equation 1, and the experimental process was repeated five times:

(1) $$S_{w}=\frac{m_{1}-m_{0}}{m_{0}}\;\times \;100\%$$

### Moisture retention experiment

The initial weight (*m_0_*) of the dry CAG fiber dressing was measured and recorded in grams. The fiber dressing was soaked in 10 ml of deionized water at a temperature of 37°C. At specific time intervals, the CAG fiber was removed and centrifuged at 5000 rpm for 15 min, excess water on the surface was absorbed using filter paper, and the weight of the centrifuged sample (*m_3_*) was measured. The moisture retention ratio (*M_r_*) was calculated using the formula shown in Equation 2. The experiment was repeated five times:

(2) $$M_{r}=\frac{m_{3}-m_{0}}{m_{0}}\;\times \;100\%$$

### Mechanical test

The mechanical properties of the hydrogel were tested using a universal testing machine equipped with a 50 N load cell. The CAG fiber sample with a length of 40 mm was clamped, and stretched at 25°C with a rate of 10 mm/min. The tensile stress (σ) was calculated by dividing the tensile load by the cross-sectional area of the fiber.

### *In vitro* release of growth factor

The dry PRP-gel and the CAG-P fibers were separately encapsulated in dialysis bags (100 k molecular weight cut-off (MWCO)), immersed in PBS, and dialyzed at 37°C. PBS (300 μl) was collected at predefined time intervals during dialysis, and an equal volume of fresh PBS was added to the system. The amount of VEGF, P-selectin and lactate dehydrogenase (LDH) in the collected PBS was detected using an enzyme-linked immuno-sorbent assay kit.

### Antibacterial performance evaluation

A 10 μl bacterial suspension was prepared, and CAG fibers were placed in the bacterial suspension and cultured in a bacterial incubator at 37°C for 24 h. Bacteria viability was assessed by staining with 100 μg/ml fluorescein diacetate and 3 μg/ml propidium iodide. The viability of bacteria was observed using a fluorescence-inverted microscope, and the bacterial death rate was calculated using Image J software.

### *In vitro* biocompatibility assay

The biocompatibility of the fiber dressings was evaluated using the Cell Counting Kit-8 (CCK-8) assay and fluorescence staining. CAG fiber and CAG-P fiber were placed in a 48-well plate and sterilized under ultraviolet light for 24 h. L929 fibroblast cells were added to each well at a density of 1.5 × 10^4^ cells per well and cultured at 37°C in a 5% CO_2_ environment. On days 1, 3, 5 and 7, the culture medium was removed from each well, and 200 μl of culture medium containing CCK-8 (CCK-8 reagent: culture medium volume ratio of 1:10) was added. After incubating in the culture incubator for 1 h, 100 μl from each well was aspirated, and the absorbance was measured at 450 nm using a microplate reader (Biotek, Synergy). L929 fibroblast cells were stained on days 1, 3, 5 and 7 to observe cell morphology on the fiber dressings. The samples were fixed with 4% paraformaldehyde, permeabilized with 1% Triton X100 for 5 min, and stained with phalloidin for 1 h and 4',6-diamidino-2-phenylindole (DAPI) for 10 min. Cell cytoplasm appeared green, and cell nuclei appeared blue under a fluorescence-inverted microscope.

### *In vivo* repair of rat skin defect

The *in vivo* experiments comply with the basic principles in Chinese Laboratory Animal—Guideline for Ethical Review of Animal Welfare (ethics number: IAC(S)202108011). A circular full-thickness skin wound measuring 1.5 cm in diameter was surgically created on the backs of 45 anesthetized rats. On postoperative day 0, the wounds were treated with gauze (negative control group), PRP-gel (positive control group), 0 d CAG-P fiber (experimental group, CAG-P fibers stored for 0 days), 90 d CAG-P fiber (experimental group, CAG-P fibers stored for 90 days), and 180 d CAG-P fiber (experimental group, CAG-P fibers stored for 180 days), each applied once. The treatments were secured with sterile dressings, with nine rats in each group. Wounds were photographed on days 0, 7, 14 and 21, and the wound closure area was calculated using Image J. The wound healing rate (*W_R_*) was calculated using Equation 3:

(3) $$W_{R}=\frac{A_{0}-A_{n}}{A_{0}}\times 100\%$$

where *A_0_* is the initial wound area on day 0 (mm^2^); *A_n_* is the wound area on day n (mm^2^).

### Histomorphology analysis

Hematoxylin-eosin (H&E) staining and Masson staining: Three rats from each group were euthanized on days 7, 14 and 21 of the experiment. Tissue samples were collected circularly around the wound (3 mm from the wound edge). These samples were then fixed with 4% paraformaldehyde, embedded in paraffin, and subsequently subjected to both H&E staining and Masson staining. Finally, the stained samples were observed under a light microscope for further analysis.

CD31 immunohistochemical staining: After submerging tissue slices in xylene and anhydrous ethanol, they were rinsed with PBS to remove any residual liquid. Following this, CD31 rat antibodies were added, and the slices were then incubated at 4°C in the dark for 24 h. After removing and sealing the tissue slices with neutral resin, they were examined under a light microscope.

### Statistical analysis

All data were acquired from at least three replicate samples and presented as mean ± standard deviation (SD). Statistical analysis was performed using one-way or two-way analysis of variance (ANOVA) with GraphPad Prism 8.0 software. Statistical significance was defined as *P <* *0.05, **P <* *0.01, ***P <* *0.001, ****P <* *0.0001.

## Results and discussion

### Microscopic structure and physical properties of CAG fibers

**Figure 2.**
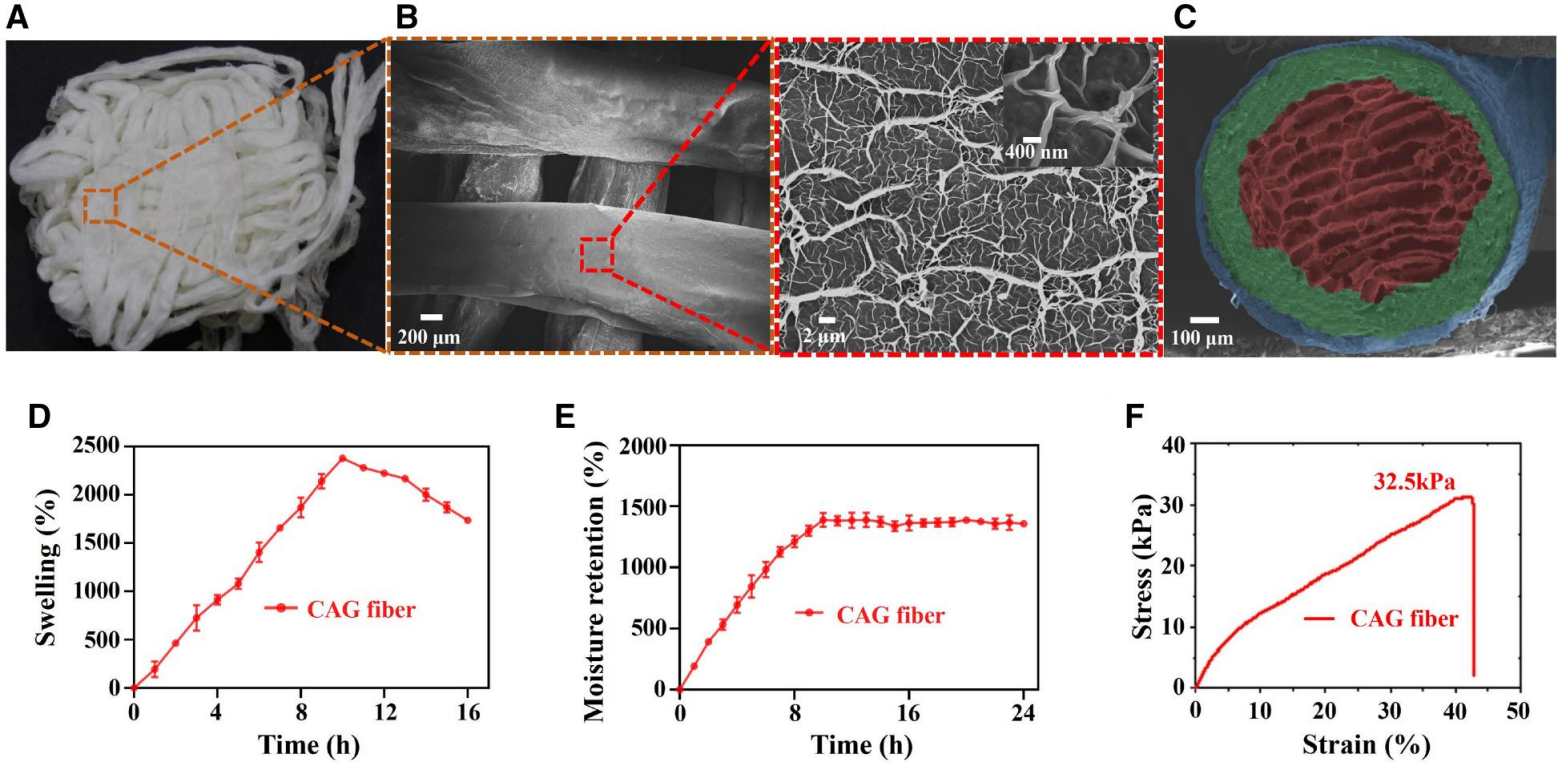
Microscopic structure and physical properties of CAG fibers. (**A**) Physical photograph and (**B**) SEM images of CAG fibers, and the surface of CAG fiber showing a disordered dendritic network structure. (**C**) SEM image showing the cross-section of CAG fiber with a triple-layered core-shell structure (each layer was pseudo-colored for contrast). (**D**) Swelling ratio (37°C, pH = 7.4), (**E**) moisture retention rate and (**F**) tensile fracture curve of CAG fibers.

Figure 2A and B shows the digital photograph and SEM images of CAG fiber dressing fabricated by a combination of one-step coaxial bioprinting and freeze-drying. It is obvious that the fiber dressings exhibit hierarchical structure. The CAG fiber dressing with a pore size of 300–600 μm could allow rapid liquid absorption of exudate and cell penetration, the fibers with a size of 500–800 μm could provide platform for cell proliferation, and the nanofibrous structures with a size of 60–1200 nm caused by dehydration could promote cell adhesion and proliferation [33]. Moreover, the fiber exhibits a distinct triple-layered core-shell structure (Figure 2C), with the outer CS layer in blue, the middle calcium alginate (CaA) layer in green, and the inner gelatin layer in red. The outer layer is thinner than the middle layer due to the weaker electrostatic complexation between CS and CaA. Notably, the innermost layer of the fiber presents a porous sponge-like structure, facilitating the absorption of a large amount of exudate from the wound. As shown in Figure 2D, the swelling ratio of CAG fiber reaches as high as 2378% after 10 h of soaking in PBS. The swelling ratio diminishes beyond 10 h of immersion due to the degradation and exfoliation of CS from the fiber surface. Additionally, the moisture retention of CAG fiber is notably high, reaching 1388% (Figure 2E), suggesting its exceptional swelling and moisturizing capabilities, making it suitable for wound care. Furthermore, for diabetics, the most common site of ulceration is the foot, where frequently consistent motion is encountered [34]. Therefore, the hydrogel dressing used must have a certain mechanical strength. The tensile stress–strain curve of CAG fiber dressing demonstrates a tensile strain of 42.8% and tensile strength of 32.5 kPa, as depicted in Figure 2F. These experimental results suggest that CAG fiber dressing prepared through freeze-drying can meet the fundamental mechanical requirements of wound dressings, exhibiting excellent moisture absorption to manage the exudate from chronic wounds such as diabetic foot ulcers. Simultaneously, the outstanding moisturizing properties of CAG fiber dressing create a conducive environment for the process of wound healing [35].

### Sustained release performance and long-term storage stability of CAG-P fibers

**Figure 3.**
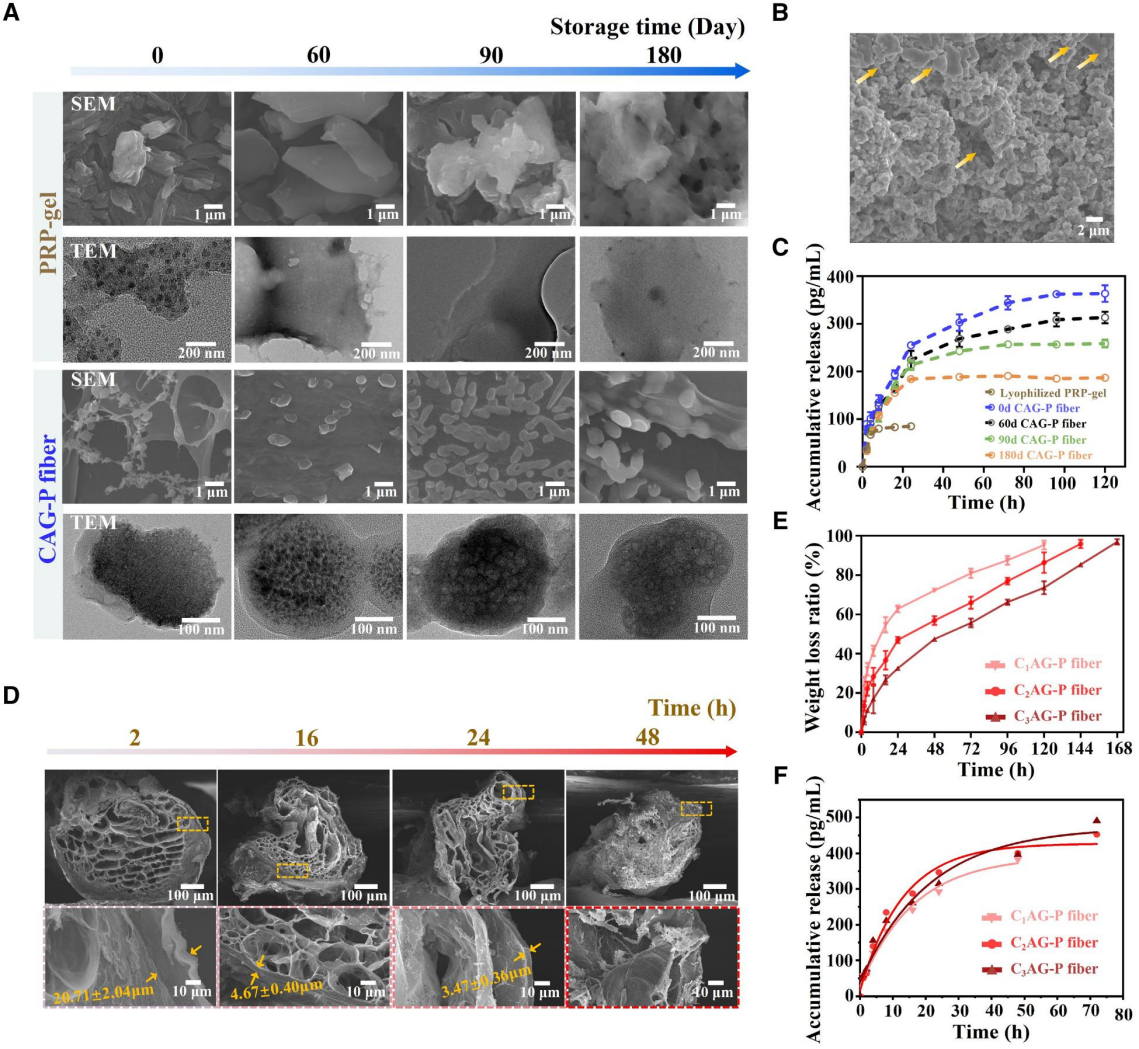
Microstructure and degradation performance of CAG-P fibers. (**A**) SEM and TEM images of PRP loaded in the freeze-dried PRP-gel and CAG-P fibers following 0, 60, 90 and 180 days of storage at −20°C. (**B**) Morphology of PRP after rehydration within the fibers following 180 days of storage (arrows indicate non-activated PRP). (**C**) Release of VEGF from freeze-dried PRP-gel and CAG-P fibers after 0, 60, 90 and 180 days of storage. (**D**) SEM images of CAG-P fibers after degradation in PBS for 2, 16, 24 and 48 h. (**E**) Degradation performance and (**F**) VEGF release performance of CAG-P fibers prepared with different outer layer flow rates (C_1_, C_2_ and C_3_ represent outer layer CS solution flow rates of 50 ml/h, 100 ml/h and 150 ml/h, respectively, and the release data were fitted by first-order kinetic model).

Platelets are small fragments of cytoplasm shed from megakaryocytes in the bone marrow. Under normal conditions, they have a biconvex discoid structure, which extends filopodia when stimulated, becoming irregular and showing significant heterogeneity. Therefore, by observing the shape of blood platelets, one can determine their activation status, which in turn helps to evaluate the long-term storage stability of PRP. To promote the long-term storage stability of PRP, PRP was loaded into the core layer of CAG fibers, thereby generating CAG-P fibers which are biologically active and allow extended storage. Freeze-dried PRP-gel served as a control group. The morphology of PRP was investigated to evaluate the function of the triple-layered core-shell structure of CAG-P fibers during storage in a vacuum environment at −20°C. Figure 3A depicts SEM and TEM images of freeze-dried PRP-gel and CAG-P fibers following 0, 60, 90 and 180 days of storage. The majority of platelets in freeze-dried PRP-gel suffer damage to their membrane structure, presenting an irregular shape. Additionally, with prolonged storage time, platelet aggregation intensifies, gradually forming a mass-like structure. TEM images also depict a gradual reduction in α-granules and dense granules within platelets as storage time increases. In contrast, platelets within CAG-P fibers are more evenly dispersed, displaying smooth circular morphologies with minimal pseudopod formation and no aggregation. Furthermore, TEM images reveal intact surface membrane structures in platelets with plentiful α-granules and dense granules. After 180 days of storage, no apparent difference was observed in platelet morphology and internal granules compared to platelets stored for 0 days, indicating that the structure of CAG-P fibers can prolong the activity of PRP. The observed phenomenon can be attributed to the presence of a dense CS and AL shell in CAG-P fibers, which shields the plasma membrane from rapid ice crystal formation during the freeze-drying process with liquid nitrogen, thus minimizing platelet deformation and death. Additionally, the AL intermediate layer within the fiber may delay ice crystal formation by lowering the temperature at which they form and promoting vitrification [36–38]. Moreover, the large molecules of gelatin in the core layer of CAG-P fibers reduce the efflux of intracellular water from platelets during the freezing process, preventing osmotic damage [39]. The porous structure of the core layer offers ample loading sites for platelets, preventing aggregation during storage, which could otherwise activate the platelets and shorten the activity of PRP. This design effectively prolongs the PRP's activity.

The activity of stored PRP can be detected by assaying the morphology of rehydrated platelets [40]. In this study, CAG-P fibers stored for 180 days were rehydrated and examined to assess the activity of platelets. As shown in Figure 3B, after rehydration and activation for 24 h, many platelets in the core layer continued to aggregate and remained activated. Additionally, some platelets appeared spherical, indicating an inactive state (yellow arrows in Figure 3B). This indicates that platelets in CAG-P fibers can be reactivated, preserving their biological activity, with only a small fraction undergoing death during storage.

VEGF is a representative growth factor present in PRP. To further validate the activity of PRP in CAG-P fibers, the release behavior of VEGF from freeze-dried PRP-gel and CAG-P fibers stored at −20°C in a vacuum environment for varying durations (0, 60, 90 and 180 days) was investigated (Figure 3C). The release of VEGF from freeze-dried PRP-gel exhibited a burst release pattern, reaching equilibrium within 8 h. Conversely, the sustained release of VEGF from CAG-P fibers extended to over four days. Notably, the cumulative release concentration of VEGF from CAG-P fibers was significantly higher than that from freeze-dried PRP-gel. The protective function of the core-shell structure of CAG-P fibers during the rigorous freeze-drying process is responsible for preserving the activity of PRP. As the storage duration increases, there is a gradual decrease in the cumulative release concentration of VEGF from CAG-P fibers, with a reduction of approximately 30% observed after 180 days of storage. Platelet storage lesions (PSLs) occurring in PRP during storage at low temperatures may account for this phenomenon. Cold-induced platelet swelling triggers a shift from a discoid to a spherical shape, prompting calcium leakage and subsequent release of α-granules and lysosomes. Ultimately, this process leads to partial platelet deactivation [41].

Based on the above results, it was hypothesized that the sustained release of GFs from CAG-P fibers is attributed to the triple-layered core-shell structure. Degradation experiments were conducted to further validate this hypothesis. After immersing CAG-P fibers in PBS for 2 h, the cross-linking bonds between CS and AL in the fibers broke, introducing gaps on the fiber surface, as depicted in Figure 3D. The rapid penetration of PBS into the fiber triggered a reaction with the AL in the middle layer, causing the AL layer to visibly disappear and reducing the thickness of the CS layer from the original 20.71 μm to 4.67 μm within 16 h. The fiber structure also deformed due to the rupture of CS and AL cross-linking bonds. By the 24-hour mark, the structure of CAG-P fibers experienced severe deformation, with the CS layer thickness reduced to only 3.47 μm. Furthermore, after 48 h, only the gelatin layer remained, and the fiber structure collapsed entirely. The results indicate that the CS layer is the key factor in the degradation of CAG-P fibers. The dense CS layer provides an advantage in resisting the entry of PBS into the fiber core, thus preventing the rehydration activation of PRP. Therefore, by regulating the flow rate of the CS layer and controlling the thickness of the outer CS layer, the degradation rate of CAG-P fibers can be controlled, subsequently regulating the release of GFs. When the flow rates in the middle and inner layers remained the same, the complete degradation time of the fiber extended from 120 h to 144 h and then 168 h as the flow rates of the CS layer increased to 50, 100 and 150 ml/h, respectively (Figure 3E). During the degradation process of CAG-P fibers, their unique triple-layered core-shell structure provides spatial resistance for platelet migration, thereby slowing down the entry of PBS into the fiber core, which further influences the release of GFs [42, 43]. Therefore, the VEGF release performance of CAG-P fibers prepared with different outer layer flow rates was tested, and the data were fitted by first-order kinetic model and showed in Figure 3F. In the first 24 h, it was found that the slope of fitting curve of C_2_AG-P group was larger than that of C_3_AG-P group, meaning the release speed of C_2_AG-P group is faster. After 24 h, the fitting curve slope of C_2_AG-P group became smaller gradually. On the contrary, the VEGF release of C_3_AG-P group still showed an increasing trend. Due to the flow rate of 50 ml/h was too slow, the C_1_AG-P fibers were quite unstable, resulting in the release finished in a short time and the total release concentration of VEGF smaller than that of other groups. All these results indicate that the dense CS layer can prevent the rehydration activation of PRP and prolong the release time of factors from PRP. Thus, the sustained release of GFs from the core can be achieved by regulating the core-shell structure of CAG-P fibers.

The aforementioned results suggest that CAG-P fibers exhibit characteristics conducive to long-term preservation and maintenance of PRP activity, while also demonstrating the capability to modulate the release of GFs from PRP.

### Optimization of freezing and storage conditions

**Figure 4.**
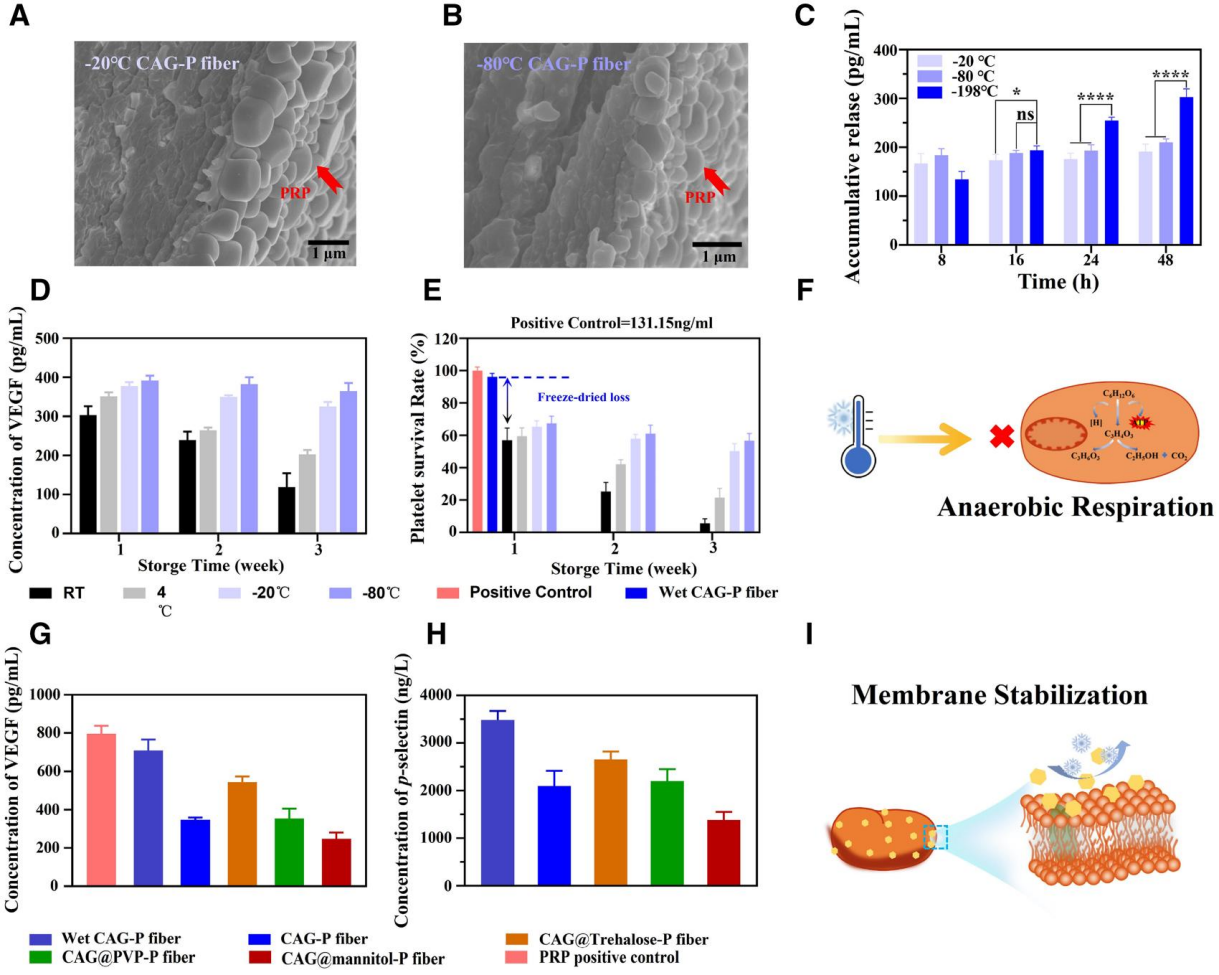
Optimization of freezing and storage conditions to enhance the storage performance of PRP. (**A**) SEM images of CAG-P fibers frozen at −20°C and (**B**) −80°C. (**C**) Cumulative release of VEGF from CAG-P fibers prepared under different freezing temperatures. (**D**) Cumulative release of VEGF and (**E**) LDH at different storage temperatures. (**F**) Schematic representation of low-temperature inhibition of platelet anaerobic respiration. (**G**) Cumulative release of VEGF and (**H**) content of *P*-selectin after PRP being treated with various lyoprotectants. (**I**) Schematic representation of the principle of lyoprotectant protection of platelets.

The rate of cooling, a critical factor in the freeze-drying process, can influence the equilibrium between osmotic pressure and contraction, as well as the formation of intracellular ice crystals. To investigate the impact of freezing rate on PRP within CAG-P fibers, wet CAG-P fibers were treated at −20°C, −80°C and −196°C (liquid nitrogen). As depicted in Figure 4A and B, CAG-P fibers treated at −20°C and −80°C collapsed, and the internal porous structure disappeared, causing PRP encapsulated inside to be squeezed and adhered to the outermost CS surface. This pronounced aggregation of platelets led to the rapid activation of PRP, with the cumulative release of VEGF reaching equilibrium within 8 h (Figure 4C), measuring 167 pg/ml and 183 pg/ml, respectively. On the contrary, rapid freezing with liquid nitrogen effectively encapsulated PRP within the fibers (Figure 3A), thereby preserving its long-term activity and enabling sustained release. The cumulative release of VEGF reached 302 pg/ml within 48 h. With the accelerated cooling rate, water is unable to traverse the chemical gradient from the intracellular space to the extracellular medium. Consequently, no significant osmotic stress or contraction is observed, and a substantial amount of intracellular water is retained, thereby preserving the activity of platelets [41, 44].

To investigate the effect of storage conditions on PRP activity, the cumulative release of VEGF and lactate dehydrogenase (LDH, a soluble cytoplasmic enzyme that is ubiquitous in cells and functions as an indicator of cell damage and necrosis [45]) from CAG-P fibers stored for 1, 2 and 3 weeks at various temperatures (room temperature, 5°C, −20°C and −80°C) was measured. As shown in Figure 4D, the cumulative release of VEGF from CAG-P fibers stored at −20°C and −80°C for three weeks remained unchanged. Conversely, after three weeks of storage at room temperature and 5°C, the cumulative release of VEGF decreased by 184.41 pg/ml and 149.03 pg/ml, respectively. The results (Figure 4E) indicate that after three weeks of storage, platelet activity in CAG-P fibers stored at room temperature and 5°C was approximately 5% and 20%, respectively, while platelet activity was about 55% at −20°C and −80°C. These findings suggest that lowering the storage temperature can mitigate PRP damage during storage. It is speculated that the low-temperature condition may inhibit anaerobic respiration in PRP, thereby reducing intracellular metabolic activity (Figure 4F). Additionally, a low-temperature environment may mitigate bacterial contamination, potentially inducing dormancy in lactate dehydrogenase (LDH) and preserving intracellular LDH activity, thereby reducing PRP loss. Upon returning to room temperature, LDH may reactivate [46, 47].

Platelets and internal proteins can be stabilized by introducing suitable protectants during freezing and drying, thereby maintaining the activity of PRP. In this study, polyvinylpyrrolidone (PVP), mannitol, and trehalose were utilized as protectants. To enable the active proteins on the surface of PRP to bind with the protectants, PRP was subjected to incubation treatments using 5 wt% solutions of each protectant. As depicted in Figure 4G, no significant difference was observed in VEGF release between wet CAG-P fibers and fresh PRP (positive control), indicating minimal damage to PRP during the preparation process. However, the VEGF release from freeze-dried CAG-P fibers was reduced by approximately 50% compared to wet fibers. The inclusion of PVP and mannitol protectants during the preparation of CAG-P fibers did not significantly increase VEGF release compared to unprotected fibers, suggesting that PVP and mannitol did not provide effective protection for PRP. Conversely, when trehalose was used, the loss in VEGF release was only about 20%. The potential reasons for trehalose exhibiting this notable protective effect are as follows; (i) Trehalose could replace water and form additional hydrogen bonds with P-selectin, a transmembrane protein. During platelet activation, P-selectin migrates from intracellular granules to the outer membrane [48], aiding in the formation of membrane lipids and thereby preventing platelet denaturation (Figure 4H); (ii) Trehalose serves as a substitute for water during the drying process, preserving the functional conformation of platelet components by creating a glassy matrix with minimal molecular mobility and temporarily suspending metabolic activity; (iii) Additionally, trehalose stabilizes platelet membrane protein components, inhibiting additional ice crystal formation and indirectly safeguarding against platelet damage during freeze-drying (Figure 4I) [48–53]. Hence, the trehalose protectant notably amplifies the protective effect on the platelet membrane surface, mitigating the loss of platelet activity during the freeze-drying process.

### *In vitro* antibacterial activity and biocompatibility of CAG-P fibers

**Figure 5.**
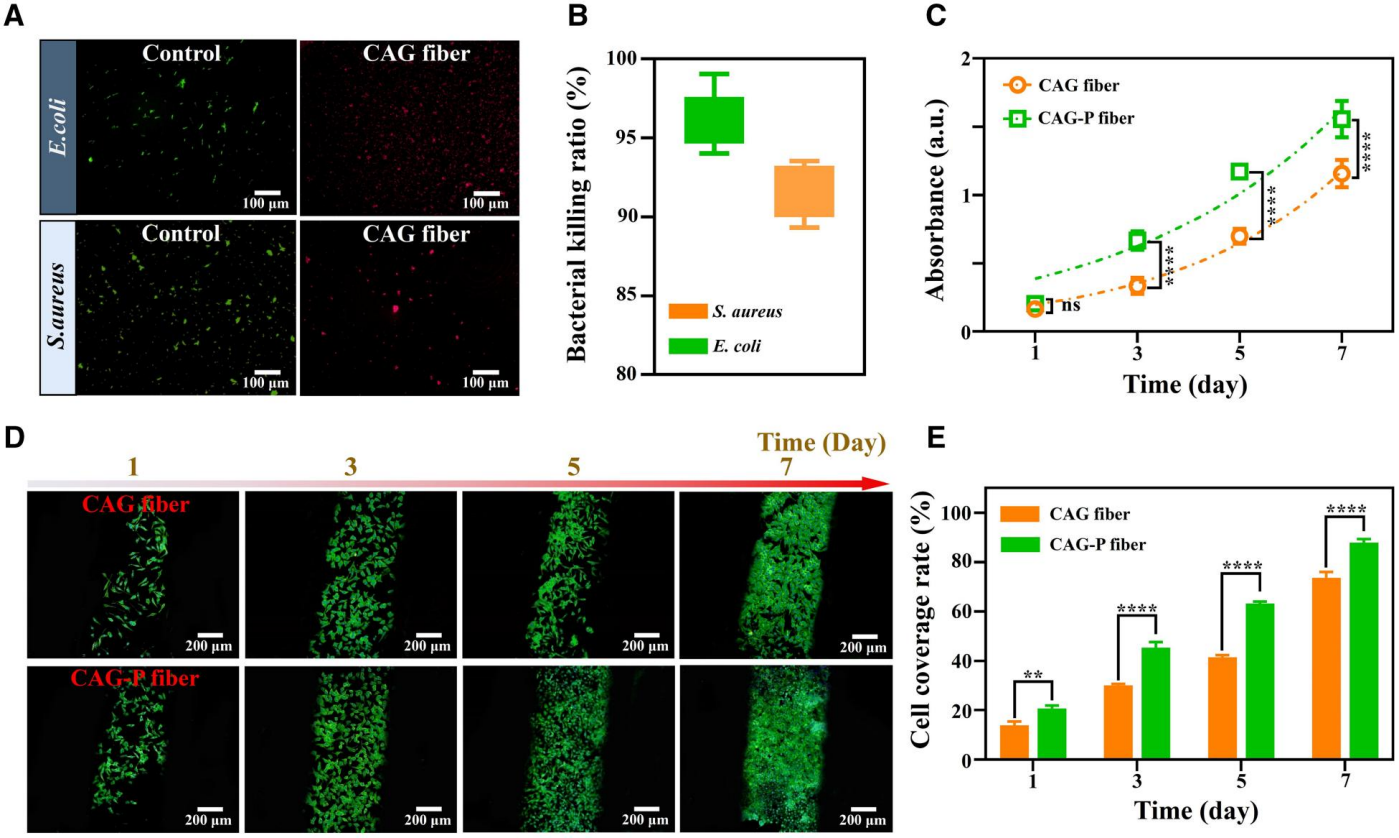
*In vitro* antibacterial performance and biocompatibility of CAG-P fibers. (**A**) Live/dead staining images of bacteria after 24 h of incubation in CAG fiber and (**B**) bactericidal rate. (**C**) Cell proliferation of L929 cells on CAG fiber and CAG-P fiber on days 1, 3, 5 and 7. (**D**) Coverage of L929 cells on CAG fiber and CAG-P fiber and (**E**) cell coverage rate. (*P < 0.05, **P < 0.01, *** *P* < 0.001, **** *P* < 0.0001).

Extended air exposure increases the susceptibility of wounds to bacterial infections, which can significantly hinder the healing process, especially in the case of chronic wounds. Hence, CAG-P fibers should exhibit specific antibacterial characteristics to maintain their activity during long-term storage. A live/dead staining method was used to evaluate the antibacterial properties of CAG-P fibers against *Escherichia coli* (*E. coli*) and *Staphylococcus aureus* (*S. aureus*). Following a 24 h incubation at 37°C, the live/dead bacterial staining results indicated that both *S. aureus* and *E. coli* displayed substantial cell mortality on CAG-P fibers (Figure 5A); the death rates for these microorganisms were 96.32% and 91.56%, as depicted in Figure 5B. The findings indicate that CAG-P fibers prevent bacterial contamination of PRP by creating a favorable antibacterial environment within the fibers during long-term storage. The antimicrobial capability of CAG-P fibers stems from the outermost CS layer. Here, the negatively charged bacterial cell membrane interacts electrostatically with the positively charged CS, leading to the dissolution of the cell wall and subsequent inactivation of pathogens [54].

The biocompatibility of CAG-P fibers is particularly crucial as a wound dressing. In this study, the biocompatibility of the fiber dressing was assessed using L929 cells as a research model, with CAG fibers serving as the control group. Cell viability was assessed using the Cell Counting Kit-8 (CCK-8) assay following co-incubation of L929 cells with the fiber scaffolds. The results demonstrated that the cell proliferation rates in all groups approached exponential growth (Figure 5C), suggesting that the biodegradable polymers utilized in the fiber dressing support L929 cell proliferation, demonstrating excellent biocompatibility. Notably, from the third day onwards, there was an obvious difference in cell activity between the two groups as the incubation duration increased. Specifically, cells exposed to CAG-P fibers demonstrated greater vitality than those in the control group. Subsequently, cell coverage on the fibers was assessed using phalloidin and DAPI staining. The results indicated that spindle-shaped L929 cells could effectively cover the fibers, and by the seventh day, the cell coverage on CAG-P fibers had reached a peak of 87.88%, which was 14.18% higher than that of the control group (Figure 5D and E). These findings suggest that the growth factors released from the PRP in the core layer of CAG-P fibers promote cell proliferation.

### Chronic wound repair

**Figure 6.**
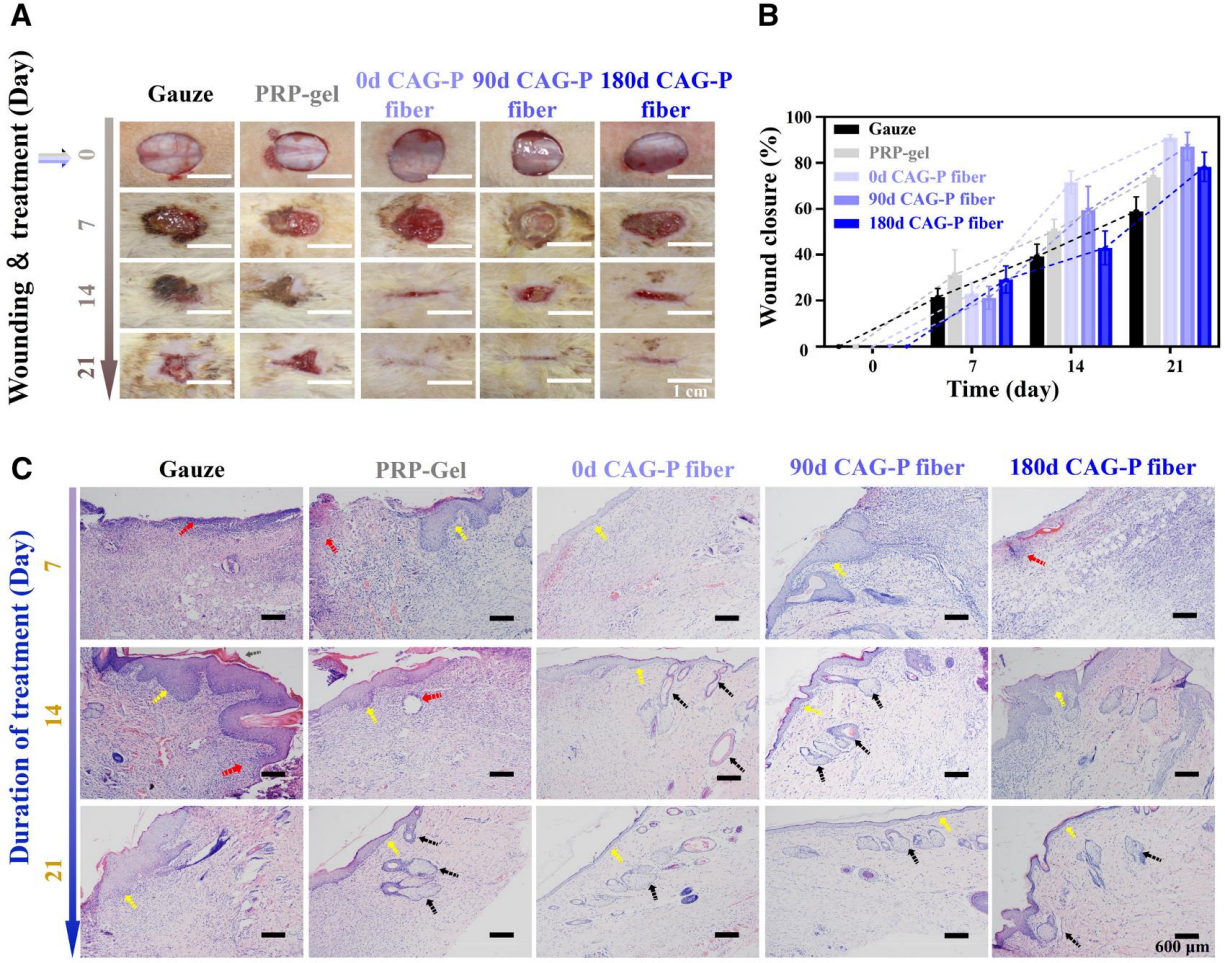
*In vivo* wound closure and healing assessment. (**A**) Digital photographs, (**B**) healing rates and (**C**) H&E stained images of wounds on days 0, 7, 14 and 21 treated with gauze, PRP-gel and CAG-P fibers after 0, 90 and 180 days of storage in SD rats (*n* = 3).

To investigate the healing ability of long-term stored CAG-P fibers on wounds, the skin regeneration capability of CAG-P fibers was evaluated using a full-thickness skin incision wound model. The gauze was used as the negative control, and clinical PRP-gel served as the positive control. A single dose was administered to the incision site on the same day when the wound model was created (day 0). After 7 days of treatment, the wound healing degree in the positive control group was higher than in other groups (Figure 6A). Notably, with extended healing time, the wound healing rates of CAG-P fibers stored for 0, 90 and 180 days gradually surpassed that of the positive control group (Figure 6B), with no observed signs of swelling or redness. These results suggest that the epithelialization process of the skin wounds was essentially completed, and wound healing entered the remodeling stage. Furthermore, a histological analysis was conducted to evaluate the healing of the incisions. Figure 6C shows the results of H&E staining. All groups exhibited acute inflammatory reactions (red arrows) in the skin tissue on the seventh day. Conversely, groups containing PRP (PRP-gel and three CAG-P fiber groups) exhibited less inflammatory infiltration. In the PRP-gel group, the epidermal layer (highlighted by yellow arrows) showed partial recovery with small scabs, indicating a better healing effect compared to the three CAG-P fiber groups. The Gauze group showed a significant presence of inflammatory cells and an incompletely recovered epidermal layer of the wound on day 14, with the latter appearing slightly thicker and only partially restored. The PRP-gel group showed slow wound healing, with the epidermal layer not fully recovered and small scabbing present, along with some inflammation. By the 21st day, the epidermal layer had fully recovered in the three CAG-P fiber groups, appearing flat, continuous and consistent, with several skin appendages such as glands and hair follicles visible (indicated by black arrows). In contrast, the epidermal layer of the Gauze group and PRP-gel group had thickened, with a small amount of inflammatory reaction still present. However, in the PRP-gel-treated skin tissue, fewer infiltrating inflammatory cells were observed, and many fibroblasts were visible in the dermis. The epidermal layer had completely recovered in all three experimental groups, with minimal cell infiltration in the dermis and the appearance of the most prominent skin appendages such as glands and hair follicles. These findings suggest that the CAG-P fiber groups demonstrated high effectiveness in wound healing.

The degree of wound epithelial healing is primarily influenced by the release behaviors of growth factors (GFs) from platelet-rich plasma (PRP). Initially, PRP-gel shows instability and is prone to rapid degradation, leading to a burst release of GFs. In contrast, the release of GFs from CAG-P fibers is linked to the degradation of the fibers, resulting in a sustained release pattern. Consequently, PRP-gel demonstrates a superior healing effect in the early stages of wound healing. However, as the healing time extends, due to the burst release, many growth factors in PRP-gel cannot be fully utilized and are lost in the wound. In contrast, the ability of CAG-P fibers to release PRP continuously can enhance its utilization and long-term therapeutic effects, thereby promoting gradual epithelialization during wound healing.

**Figure 7.**
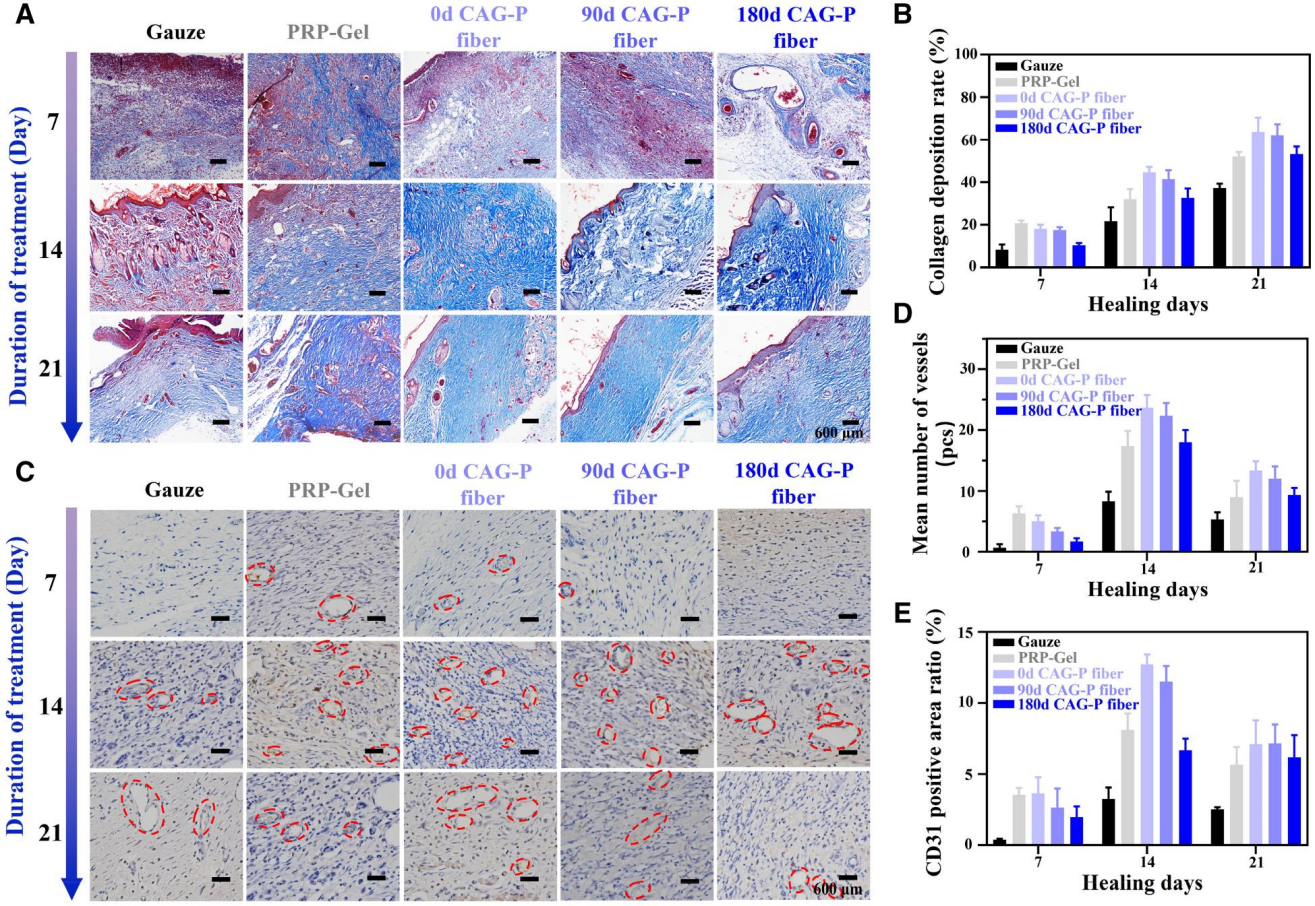
(**A**) Masson staining images of wound sections on days 7, 14 and 21 for the gauze group, PRP-gel group and CAG-P fiber treatment groups stored for 0, 90 and 180 days in SD rats; (**B**) deposition rate of collagen fibers; (**C**) immunohistochemical staining images of CD31; (**D**) average vascular quantity calculated from wound sections based on immunohistochemical staining. (**E**) Positive area ratio of CD31.

Furthermore, Masson staining was carried out to assess collagen deposition and tissue healing in different treatment groups (Figure 7A). The blue area represents collagen, while the red area represents keratin or muscle fibers. The results revealed that on the 7th day, the skin tissue in the positive control group exhibited partial collagen fibers, although they appeared immature and loosely structured. Surprisingly, the positive control group displayed a more favorable healing effect compared to the experimental group. By the 21st day, the collagen fiber content gradually increased in all groups, with higher proportions observed in the groups of CAG-P fibers stored for 0 and 90 days (Figure 7B). Notably, in these groups, the collagen fibers exhibited a more mature phenotype, characterized by a dense, tightly packed, regular, and ordered collagen network, aligned with the direction of the incision. These findings suggest that CAG-P fibers facilitate the rapid healing of full-thickness wounds.

Additionally, immunohistochemical staining of CD31 was performed to assess vascular formation at the injury site, providing insights into the efficacy of wound healing. CD31, a transmembrane protein, plays a pivotal role in cell adhesion and acts as a signaling molecule that governs angiogenesis [55]. As illustrated in Figure 7C, positive endothelial cells contributed to the formation of blood vessels, and the vascular density in the wound tissues of each group was quantified (Figure 7D) to assess the angiogenic effects across groups. On the 7th day, the PRP-gel group showed a higher vascular density compared to other groups. However, with prolonged treatment duration, the vascular density of the three CAG-P fiber groups gradually exceeded that of the control groups. This increased capillary density facilitates efficient blood flow to the wound site, thereby promoting rapid vascular regeneration during the angiogenic proliferation phase. The CD31-positive area ratio of the three CAG-P fiber groups decreased by the 21st day compared to the 14th day (Figure 7E). This observation marks the initiation of vascular regression on the 21st day, aligning with the phase of vascular inhibition and remodeling in wound healing. These findings likely stem from the sustained release behavior exhibited by the CAG-P fiber groups, wherein the cumulative release of growth factors continuously facilitated vascular formation. As a result, this process accelerated the transition from the angiogenic proliferation phase to the vascular maturation phase. This finding is consistent with the outcomes of *in vitro* experiments that demonstrate the sustained release of VEGF from the CAG-P fiber groups.

## Conclusion

In summary, a multifunctional triple-layered core-shell fiber dressing was developed through coaxial cryogenic 3D bioprinting combined with freeze-drying. The experimental results indicate that by modifying the core-shell structure of the fibers, the resulting dressing can modulate the release profile of growth factors from platelet-rich plasma (PRP). The dressing preserves the high activity and wound healing potential of PRP even after 180 days of cryogenic storage post-freeze-drying, with only a 30% decrease in cumulative VEGF release due to the protective core-shell structure. It effectively alleviates inflammation and closes skin incisions. Moreover, employing liquid nitrogen freezing, incorporating trehalose protectant, and storing at −80°C can further minimize the inactivation rate of PRP during freezing and storage procedures. Hence, CAG-P fiber enables prolonged storage and maintenance of PRP’s biological activity, along with sustained release of GFs, contributing a positive role in wound healing. This study holds substantial implications for developing biologically active medical dressings.
